# Revisiting right anterior oblique projections for the triangle of Koch: implications from computed tomography

**DOI:** 10.1186/s12872-020-01632-9

**Published:** 2020-08-24

**Authors:** Yanjing Wang, Lin Liu, Robert Lakin, Nazari Polidovitch, Guohui Liu, Hongliang Yang, Ming Yu, Mingzhou Yan, Dong Zhao, Peter H. Backx, Huan Sun, Yuquan He, Ping Yang

**Affiliations:** 1 Radiology Department, China-Japan Union Hospital of Jilin University, 126 Xiantai street, Changchun, Jilin Province 130033 China; 2 Department of Biology, York University, 4700 Keele Street, Toronto, ON Canada; 3 Cardiology Department, China-Japan Union Hospital of Jilin University, 126 Xiantai Street, Changchun, Jilin Province 130033 China; 4 Jilin Provincial Precision Medicine Key Laboratory for Cardiovascular Genetic Diagnosis, Changchun, Jilin Province 130033 China; 5 Jilin Provincial Cardiovascular Research Institute, Changchun, 130033 China

**Keywords:** Ablation, Anatomy, Triangle of Koch’s, AVNRT, Cardiac CT

## Abstract

**Background:**

Variability in the anatomy and orientation of the triangle of Koch (TK) complicates ablation procedures involving the atrioventricular (AV) node. We used CT angiography (CTA) to assess the anatomical TK orientation, the CS ostium direction, and the relationship between the two, and we validated an individualized CS-guided projection during ablation procedures.

**Methods:**

In 104 patients without structural heart disease undergoing computed tomography (CT) angiography, TK orientations were determined in relation to the coronary sinus ostium (CSo) as well as two standard right anterior oblique (RAO) projection angles (30^o^ and 45^o^) commonly used in ablation procedures.

**Results:**

A CS-guided RAO projection (RAOCS) was shown to best track the orientation of the TK compared to RAO30° and 45°, with TK orientation strongly correlating with the CSo direction (*r* = 0.86, *P* < 0.001). In addition, the mean relative difference between the angle of the CSo and TK orientation was 5.54 ± 0.48°, consistent with a reduction in the degree of image shortening compared to traditional RAOs. Moreover, in vivo validation following ablation revealed that using a CS-guided projection limited the degree of on-screen image shortening compared to both the RAO30° and 45° in 25 patients with catheter ablation procedures.

**Conclusion:**

In hearts with a normal structure, the CSo direction offers a reliable predictor of the TK orientation which can be used to guide the projection of the TK during ablation procedures.

## Background

Understanding the anatomical structure of the Triangle of Koch (TK) is critical for electrophysiologists, especially for ablation procedures involving the atrioventricular node (AVN) such as atrioventricular node reentry tachycardia (AVNRT) [1–3]. Current methods for estimating the TK anatomy during electrophysiology (EP) studies routinely use fluoroscopy images generated at right anterior oblique (RAO) projection angles of 30° or 45°, which may poorly align with the TK orientation and thereby limit spatial resolution [3], cause image shortening and then may influence the precise localization of the catheter. Unfortunately, the anatomic relationship of the TK to the other cardiac structures is highly variable between patients [4]. Thereby, taking a fixed RAO projection in ablation would potentially complicate ablation procedures [5].

Although, the 3D-mapping technology and intracardiac ultrasound can accurately visualize anatomical structures related to the TK [6, 7], these methods are costly and limited to a few centers, which make the use of traditional 2D-fluoroscope-guided ablation preferable in many centers. Thus, revisiting and optimizing traditional methods for imaging the TK may result in more accurate and individualized fluoroscope imaging approaches to overcome the influence caused by anatomical variability of the TK on procedural success. However, such an approach using more traditional technologies has yet to be validated.

As post-mortem assessments of the heart have established that the coronary sinus ostium (CSo) sits at the base of the TK [4], some electrophysiologists routinely use the CSo to locate the slow pathway within the lower region of the TK. Despite the well-known anatomical relationship between the TK and CSo in cadavers [8], no previous studies have explored the relationship between the TK and the CSo in patients referred for ablation. Hence, we used CT angiography (CTA) to assess the anatomical TK orientation, the CS ostium direction, and the relationship between the two in patients with structurally normal hearts. In addition, we sought to validate whether an individualized CS-guided projection can improve the projection by decreasing the shortening of images during ablation procedures.

## Methods

### Computerized tomography (CT) data and its screening

Three hundred ninety-nine consecutive in-patients, who underwent coronary CT scanning between January 2014 and March 2015, were screened. After assessment, 295 patients were excluded as following: prior heart surgery history (*n* = 15), significant enlargement of heart chambers (*n* = 25), congenital heart diseases (*n* = 9), coronary artery stenosis with clinical significances(defined as a stenosis over 50%; *n* = 234), or technical issues resulting in obscured imaging of the TK or the CS (*n* = 12). The remaining 104 patients’ CT data were included in our study. To avoid observer bias, the screening and analysis of CT data were performed by two independent radiologists with an experience over 4 years in interpreting cardiovascular imaging. The results were double-check and confirmed by a senior radiologist with a 10-year experience in interpretation of cardiovascular imaging. This study was performed according to the World Medical Association Declaration of Helsinki, and it was approved by the Ethics Committee of China Japan Union Hospital of Jilin University (decision number: 2019082109).

### Protocol used for CT scan

A heart rate of < 65 beats/min was used as the target heart rate in order to ensure accurate image capture and proper visualization. Oral metoprolol was given to achieve the target heart rate, if necessary. The heart rate ranged 58–68 beats/min immediately before CT scanning.

CT exams were performed by a 320-section multi–detector row CT scanner (Aquilion; Toshiba America Medical Systems) [9] with the aid of iohexol (Isovue 370; Bracco Diagnostics, Inc) to enhance contrast (injected at 4–5 mL/sec into the antecubital vein). Scanning parameters were set as following: number of detector rows: 320; table feed per rotation: 7.2 mm; section thickness: 0.5 mm; tube voltage: 120 kVp; tube current: 400 mA; gantry rotation time, 400 msec. During a single breath hold, a retrospective electrocardiograph-gate volumetric data set was collected. We performed multi-segment reconstruction using diastolic imaging of the heart, which was determined by a relative-delay strategy (75% R-R interval).

### Analysis and measurements of CT data

**Fig. 1:**
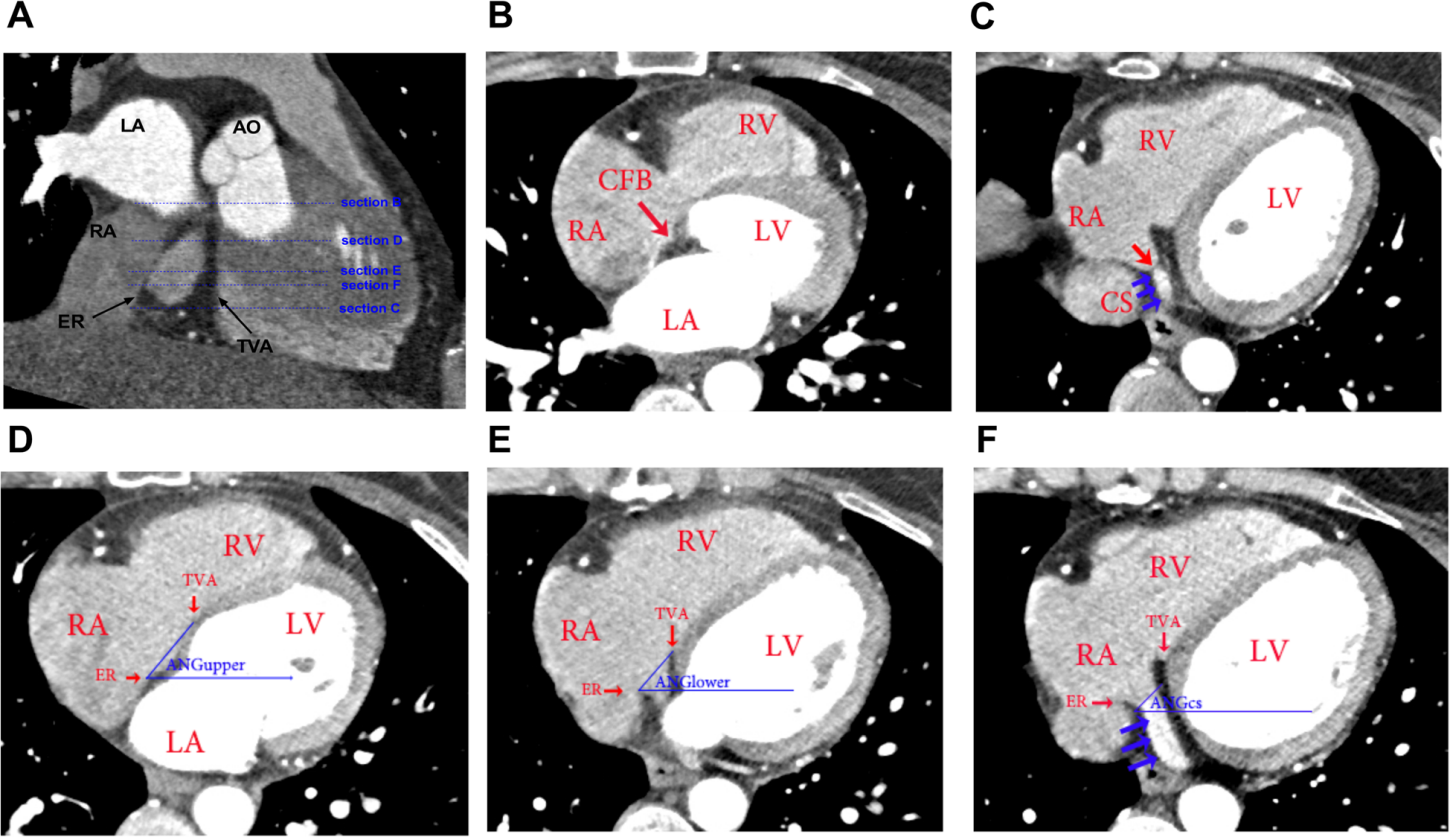
**a**-**f** Determination of Orientation of the Triangle of Koch (TK) and coronary sinus ostium (CSo) using CT imaging. **a** A multiplanar reformation CT image of the TK based on right atrial anatomical landmarks. Cross-sections (blue dotted lines labelled with Sections **b**-**f**) represent to typical CT axial imaging planes used to analyze TK (upper and lower) and CSo orientations, with the corresponding images shown in figures b-f. **b** Apical border of the TK visualized in the axial plane by the appearance of the CFB (red arrow). **c** Lower border of the TK identified in the axial plane by the appearance of the CSo bottom (red arrow), which separates the right atrium (RA) and CS (blue arrows). **d** and **e** Upper and lower TK sections. The orientation of the upper (ANGupper) and lower (ANGlower) TK is determined by the angle between the line connecting the anterior (ER) and posterior (TVA) boundaries of the triangle and a line drawn to the horizontal plane (blue lines). **f** CS section. The direction of the CSo (ANGCS) is determined by the angle between the CSo, which is determined by the line connection the anterior and posterior boundaries of the CSo, and the horizontal plane. The blue arrows show the course of the CS. AO, aorta; CFB, central fibrous body; ER, Eutachian ridge; LA, left atrium; RA, right atrium; RV, right ventricle; TVA, tricuspid valve attachment

The TK boundaries were identified in CT images using the anatomical landmarks illustrated in Fig. 1. Briefly, the apex of the TK was identified immediately beneath the aorta (AO) from which emerges the anterior boundary demarcated by the attachment of septal leaflet of the tricuspid valve and the posterior border formed by the Eutachian ridge (ER). The base of the TK is determined by the CS ostium (CSo) bottom. These anatomical landmarks were then used to identify the anatomy of the TK from the various planar slices of the CT images.

Since the TK surface may have a finite curvature, 2-dimensional planar images of the entire TK are generally not attainable. Therefore, as illustrated in Fig. 1a, we separately determined the orientation of upper (ANG_upper_) and lower (ANG_lower_) regions of the TK by estimating the angle of the line that connected the anterior tricuspid valve attachment (TVA) with the posterior portion of the ER relative to the horizontal plane of the CT bed. In addition, we also separately estimated the angle between the CSo, which is represented by the line connecting the anterior and posterior CSo boundaries, and the horizontal plane of the CT bed. Specifically, Fig. 1d-e show the representative sections used to determine the upper and lower TK orientation as well as CS directions. The CT image boundary and interpretation has been reported by Saremi F. et al. [10].

**Fig. 2:**
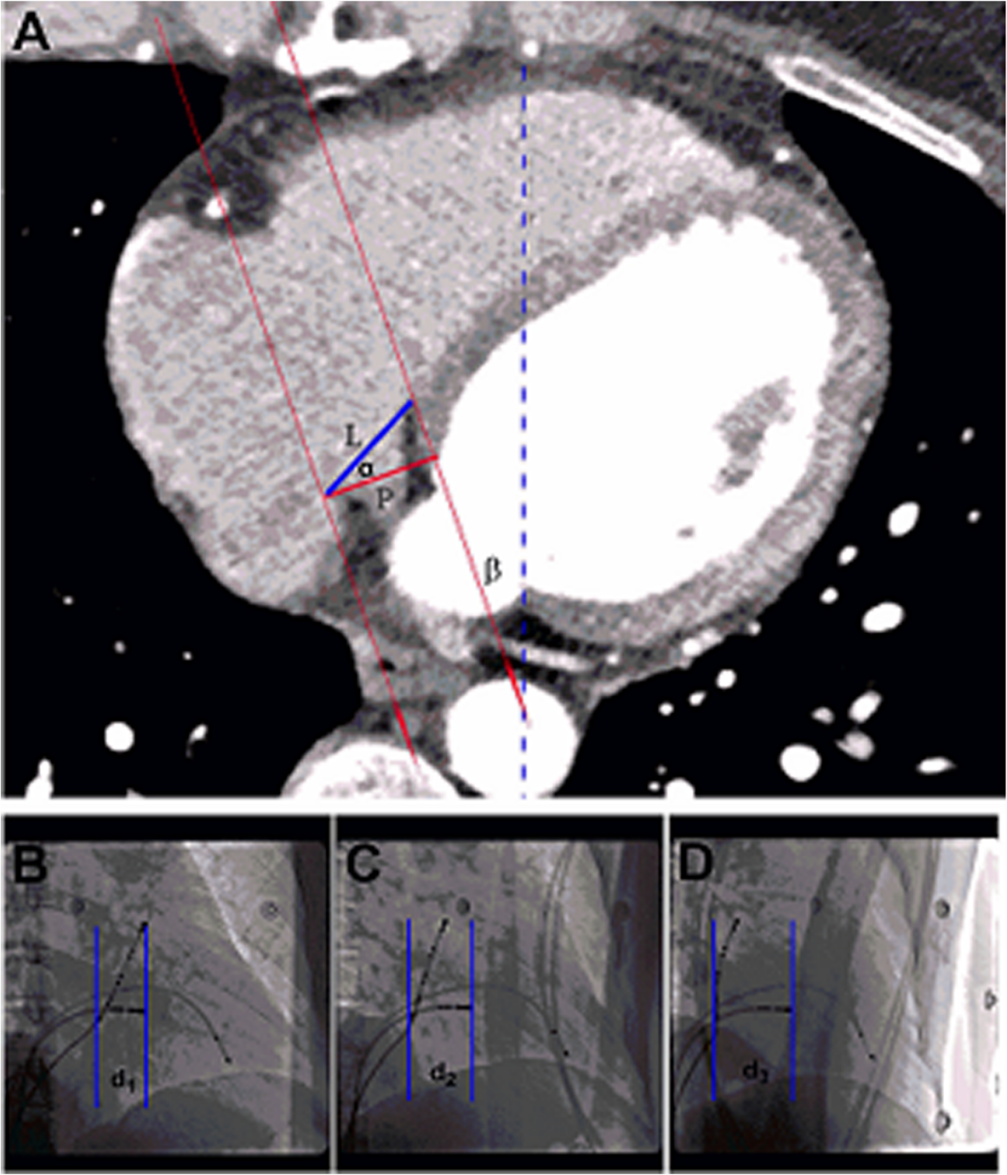
**a**-**d** Estimating the shortening rate of the projection. **a** From a given CT scanned section, the blue line showed the actual length of TK (blue line) and the X-direction (red arrowed lines) can be determined. Note that the X-ray is not enfacing the TK with a shortened projection (red line). The blue dashed line illustrates the standard line vertical to the exam bed. The angle between the red lines and the dashed blue line equals to the projection angle (the angle β). The actual TK length in a given section (L) and the estimated projection length (P) are shown. The shortening rate can be calculated by (1-P/L) × 100%. **b** through **d** Changing the projection angle influenced the image and the projection of the TK using X-ray imaging. For each projection angle used, namely RAO30 (**b**), RAO45 (**c**) and RAOCS (**d**), (see text for detail), the distances (shown by the two blue lines, and labeled by the labels d1-d3) between proximal coronary sinus (CS) and ablation catheter (ABL), the degree of image shortening was determined. RVA: right ventricle apex, CS: coronary sinus, ABL: ablation catheter

Next, we estimated the projection length on lower TK section to figure out the influence of projection angle on image gained by fluoroscope. When the X-ray was projected with an RAO30° or 45° angle to the vertical line, we obtained the image of the heart or TK, as shown in Fig. 2. Based on the projection, the rate of TK image shortening by the fluoroscope could be estimated using the following formula:

$$ \left(\mathrm{L}-\mathrm{P}\right)/\mathrm{L}\times 100\%=\left(1-\mathrm{P}/\mathrm{L}\right)\times 100\% $$

Where L is the actual length of the TK and P is the estimated projection length. A higher shortening rate would imply a more obvious shortened image. This rate of P/L is actually equals to the value of cosα (shown in Fig. 2), and was calculated by cosα.

### In-vivo validation of CS-guided projections

In order to validate whether the X-ray CS-guided RAO projection (RAOCS) limits the degree of TK image shortening during EP study and ablation procedures, we compared the distances between two given anatomical points, the CS ostium and the slow pathway, in on-screen TK projections when the RAO projection angle was changed. In addition, the two anatomical points were labeled in vivo using the ablation catheters during the procedure (Fig. 2), and the degree of image shortening calculated as discussed above.

Specifically, a total of 25 patients (11 men, 14 women) undergoing EP study and ablation for atrial fibrillation (4/25), accessory pathway (10/25), and AVNRT (11/25) from April 2018 to May 2018 were included. Following successful ablation of the original arrhythmia, we positioned the mapping catheter at the tricuspid annulus near the ostium of the coronary sinus, which was determined by local electrograph recording a fraction A wave and an A:V ratio between 1:10 and 1:1. A 10-polar CS catheter was placed inside the coronary sinus with the most proximal electrode at the CS ostium. For those in whom the CS catheter could not be advanced deep enough, the electrode located at the CS ostium was used as a marker for CS ostium. The horizontal distances between the CS ostium and the slow pathway were measured using the RAO30, RAO45, and RAOCS projections and analyzed with a fixed catheter position (Fig. 2b-d). In particular, the RAOCS was determined by overlap of the proximal electrodes on the screen and confirmed when the projection resulted in the X-ray being perpendicular to the CS ostium.

### Statistical analysis

Data are reported as mean ± standard error (SEM). Differences between estimated means were determined (when appropriate) using t-tests. To determine whether our results were normally distributed, we applied the Kolmogorov-Smirnov (K-S) test (*P* > 0.05). The relationships between the orientation of the upper and lower TK as well as the TK and CS ostium directions were determined using Pearson’s correlation coefficients (r) with least squares regression analyses with between group comparisons made using the Bland-Altman plot [11]. All data were analyzed using SPSS 22 (IBM Inc., Armonk, NY, USA).

## Results

**Fig. 3:**
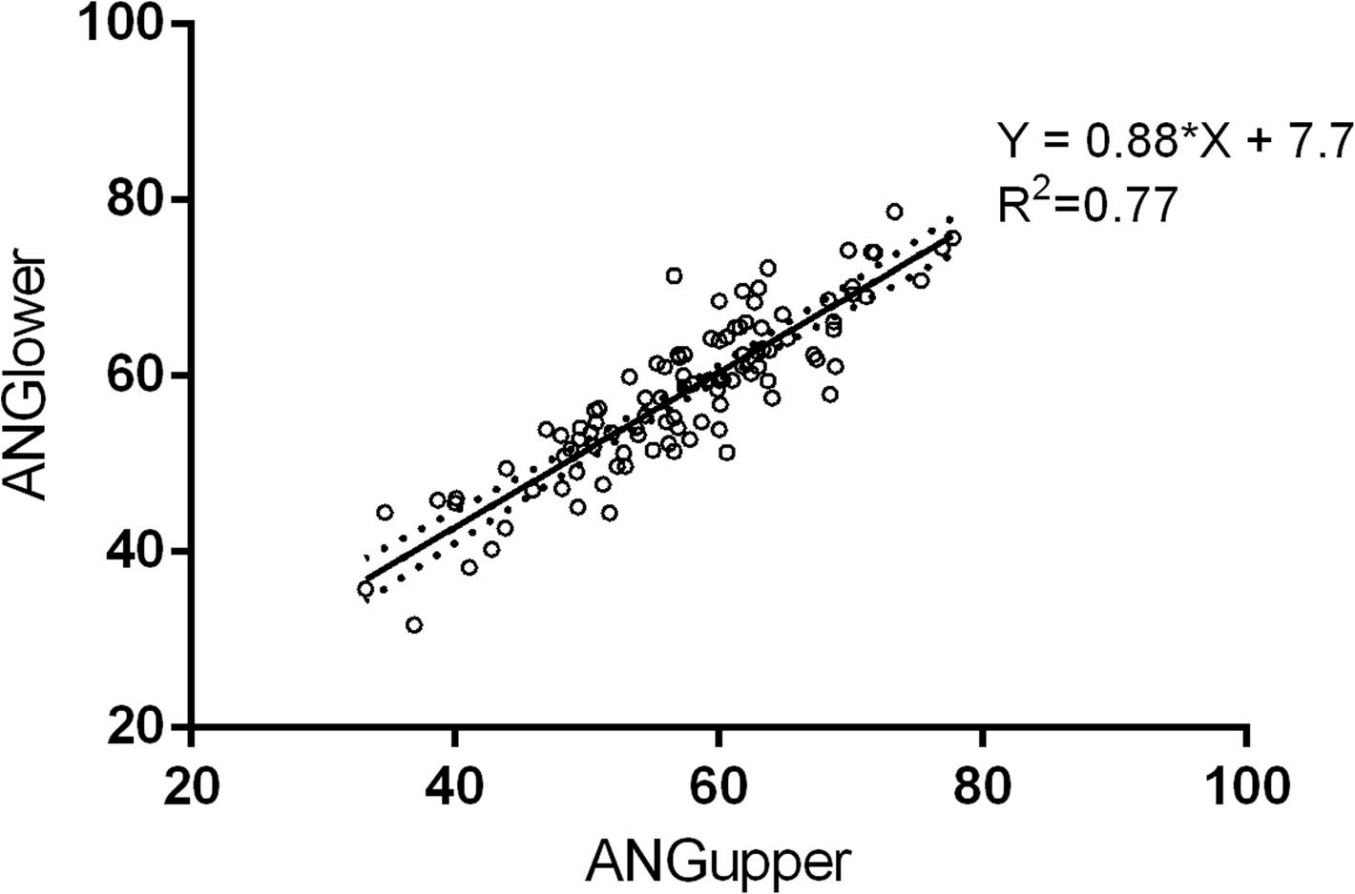
Scatter plot of the association between changes in lower (ANGlower) and upper (ANGupper) TK orientation. Linear regression analysis demonstrated a significant correlation between ANGlower and ANGupper for each patient (R2 = 0.77, *p* < 0.0001), in spite of a highly variable relationship between patients (i.e. 31.7–78.7°). Dashed lines indicated 95% confidence interval

Fifty-seven males and 47 females were included, finally, in our study. The age of these patients was 52.3 ± 8.0 years ranging 31–69 years. The screening was illustrated by the flow chart shown in Supplementary Figure 1. We began by assessing anatomical variability within the TK by determining the orientation angles of the lower and upper TK relative to the horizontal plane (ANG_upper_ and ANG_lower_) from our CT scans. This was accomplished by first identifying the CT images that maximize the area of the TK. Next, the lines connecting the anterior tricuspid valve attachment (TVA) with the posterior Eutachian ridge (ER) in axial slices that captured the upper 1/3 and lower 1/3 TK regions were determined from which the angle of the TK relative to the horizontal plane was estimated (i.e. ANG_upper_ and ANG_lower_) (Fig. 1). The results revealed that ANG_upper_ and ANG_lower_ were normally distributed (Table 1). Plots of ANG_lower_ versus ANG_upper_ for each patient (Fig. 3) established that ANG_upper_ and ANG_lower_ were strongly correlated (*r* = 0.88, *R*^*2*^ = 0.77, *p* < 0.0001) within a given patient with the no differences (*P* = 0.11) between ANG_lower_ and ANG_upper_. Thus, this indicates that the TK can be considered an untwisted structure, and we can expect a common projection angle to obtain an image with minimized shortening independently of which TK region (upper or lower) is tracked. We then used the ANG_lower_ to represent the TK orientation, as the lower TK is often used as targets of AVNRT ablation. In the patients younger than 45 years old, the TK orientation is 64.05 ± 8.39°, while the TK orientation in the patients older than 45 years is 56.76 ± 8.78°(*P* = 0.0011). The TK orientations in the male and the female are not statistically different (*P* = 0.25), shown in Supplementary Figure 2.

**Table 1:** Orientations of the Triangle of Koch and coronary sinus ostium

Variable	Range	Mean Angle
ANG_upper_	33.2–77.7°	57.2 ± 0.93°
ANG_lower_	31.7–78.7°	58.2 ± 0.89°
ANG_CS_	28.7–71.0°	52.3 ± 0.82°

**Fig. 4:**
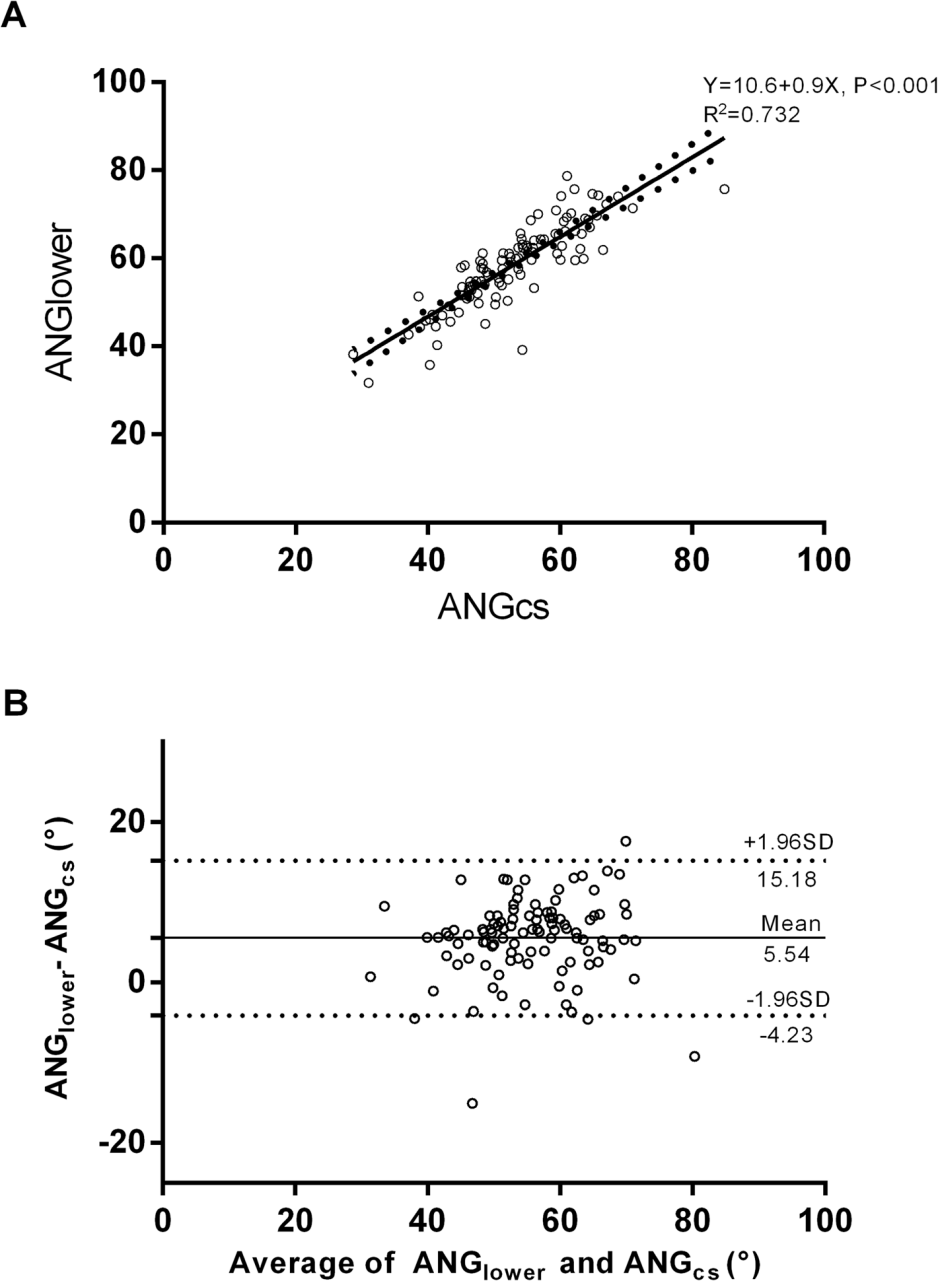
**a**, **b** Relationship between lower TK orientation and CS ostium direction. **a** Scatter plot of the association between changes in lower TK orientation (ANGlower) and CS ostium direction (ANGcs). Linear regression analysis demonstrated a significant correlation between TK orientation and CS ostium direction. Dashed lines indicated 95% confidence interval. Pearson’s correlation coefficient *r* = 0.86 (*P* < 0.001), and *R2* = 0.732. TK = triangle of Koch; CS = coronary sinus. **b** The relationship between the difference between ANGCS and ANGlower (ANGlower-ANGCS) and the average of ANGCS and ANGlower by Bland-Altman analysis. The data reveals that ANGlower-ANGCS was 5.54 ± 0.48° with the 95% confidence limits spanning from − 4.23° to 15.18° (upper and lower dotted lines)

Next, we compared the ability of two standard RAO projection angles (RAO30° and 45°) as well as a CS-guided projection (RAOCS) to track the alignment of the lower TK. The average angles for RAO30 and RAO45 relative to the lower TK were 28.8 ± 0.91° and 13.2 ± 0.91°, respectively. Linear regression analysis showed a strong correlation (*r* = 0.86, *R2* = 0.732, *P* < 0.001) between ANG_lower_ and ANG_CS_ (Fig. 4a), with TK orientation equal to 10.6 + 0.9 x CS ostium direction. Meanwhile, the angle between RAOCS and the lower TK was reduced (5.54 ± 0.48°) (*P* < 0.0001) compared to RAO30° and 45°, with the 95% limits of agreement between − 4.23° and 15.18° (Fig. 4b). Taken together, these findings suggest a CS-guided projection more closely aligns with TK orientation.

**Fig. 5:**
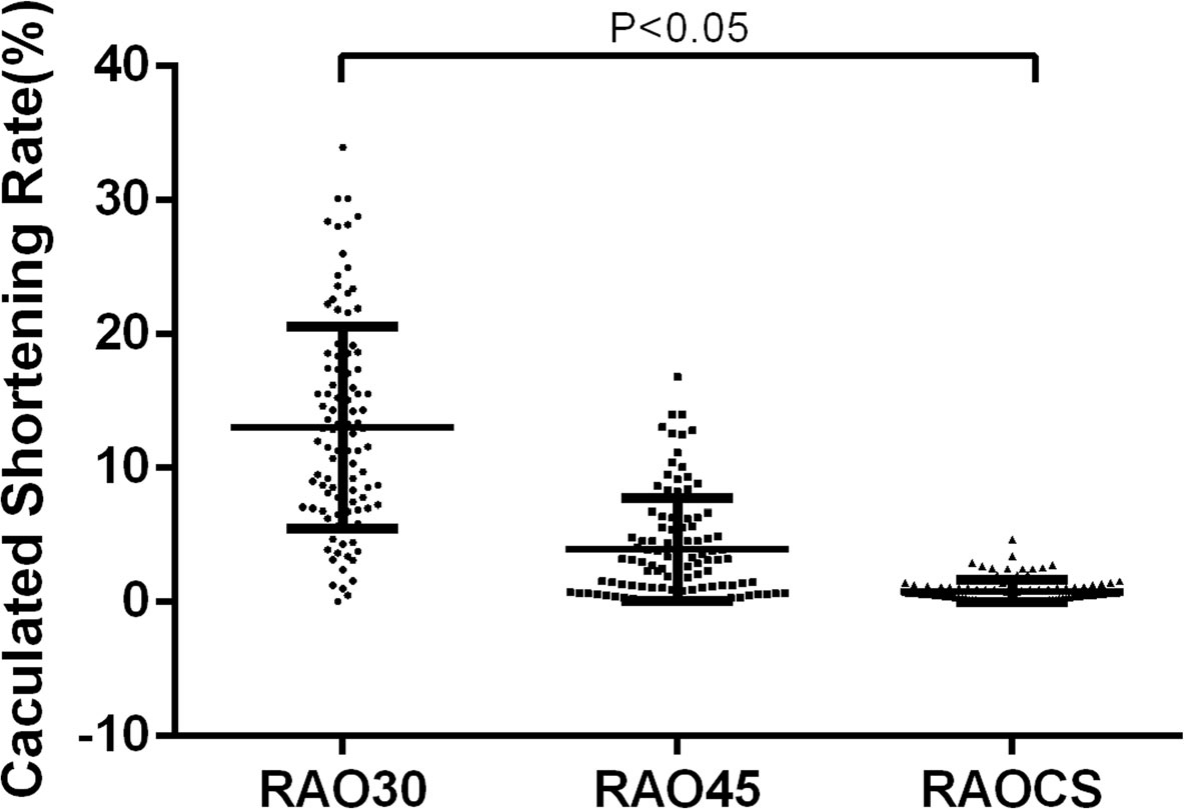
The estimated image shortening of the RAO projections with a projection angle equaling to 30° (RAO30), 45° (RAO45) and coronary sinus direction (RAOCS). The shortening rate distributions under different RAO projections are shown by plots. It can be figured out that RAOCS rendered less shortening (0.83 ± 0.84%) comparing to RAO30 (13.03 ± 7.56%) and RAO45 (3.93 ± 3.84%). *P < 0.0001. Mean ± SD as indicated

To confirm the utility of using a CS-guided projection compared to traditional RAO projection angles, we compared the estimated shortening rates of the TK image (Fig. 5). Using the RAO30° and RAO45° projections, shortening rates of 13.03 ± 0.73%, and 3.93 ± 0.37% were observed, respectively. By contrast, the degree of image shortening was reduced (0.83 ± 0.08%) (*P* < 0.05) compared to traditional RAO projection angles.

**Fig. 6:**
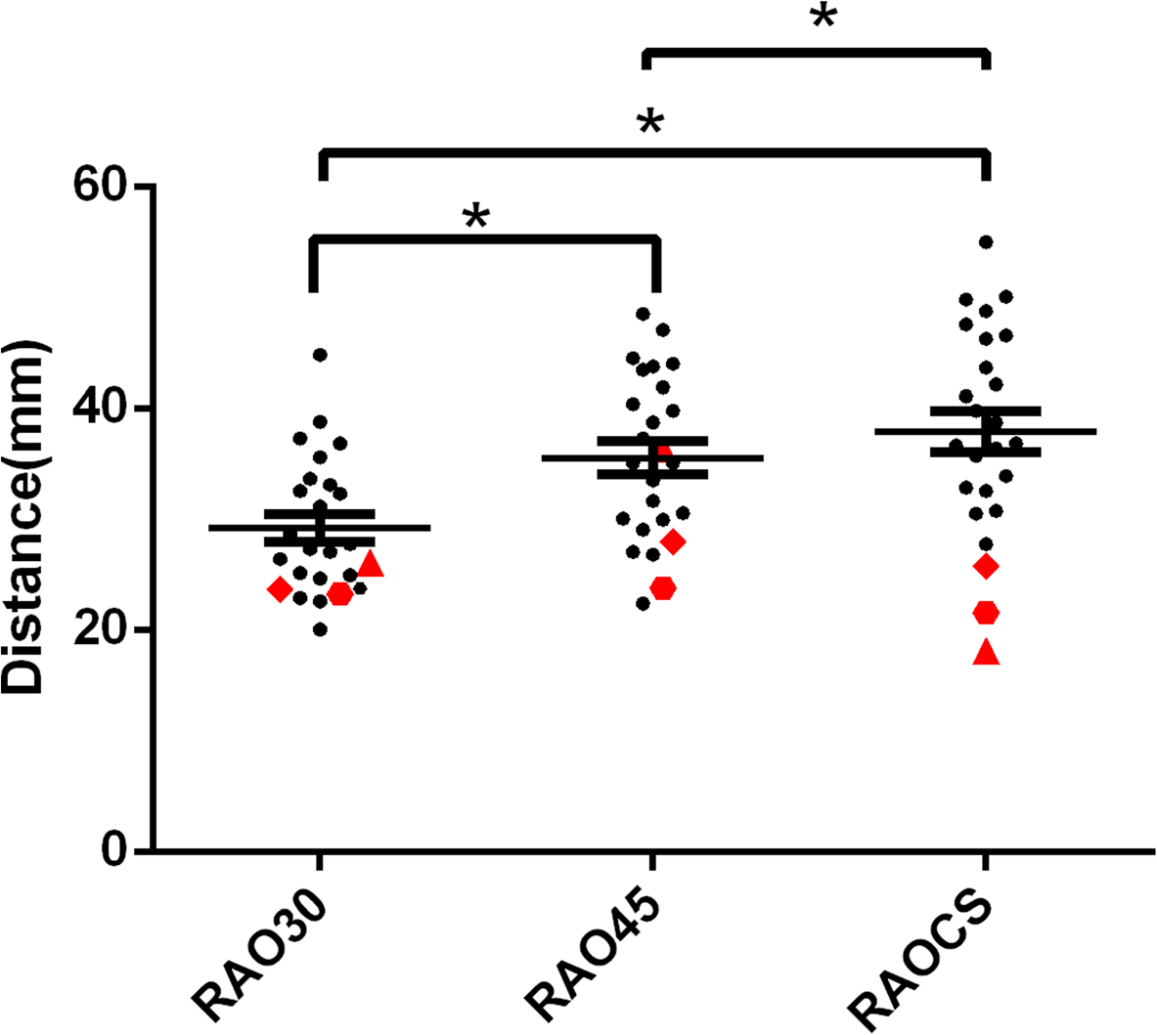
Distances between proximal CS catheter and distal ablation catheter with different projections. Three patients’ were identified as outliers (shown in red) presenting with smaller distances under RAOCS projection *P < 0.001

To further validate our findings, we sought to determine the impact of using a CS-guided projection angle (RAOCS) in vivo on TK image shortening and validate its use in a subset (*n* = 25) of patients undergoing an atrial arrhythmia ablation procedure. In analyzing the distance between the proximal electrode of the CS catheter and the distal electrode of the mapping catheter, the distances varied based on the projection angle used, with RAO30, RAO45, and RAOCS resulting in mean distances of 29.01 ± 1.19 mm, 34.99 ± 1.53 mm, 37.41 ± 1.87 mm (*P* < 0.0001), shown in Fig. 6 respectively. The average RAOCS projection angle was 51.66 ± 7.76°. In most individuals, using the RAOCS projection resulted in a larger measured distance value compared to the two traditional projections. In reviewing the RAOCS data, three patients were identified as outliners. However, in each of these outlier patients in which the CS guided projection did not lead to a smaller shortening, we found it to reflect either an anatomical or procedural issues, as listed in Table 2.

**Table 2:** Data from patients showing smaller distances under CS-guided RAO projection

Patient NO	Distance under RAO 30	Distance under RAO 45	Distance under RAOCS	Possible Reason
**1**	**26.06**	**36.35**	**18.11**	**CS catheter cannot advance to distal CS**
**2**	**23.6**	**27.95**	**25.72**	**CS catheter cannot advance to distal CS**
**3**	**23.2**	**23.75**	**21.52**	**CS locates on atrial side.**

## Discussion

Tremendous anatomical variability has been observed in TK orientation and its landmarks within patient population [11], with differences in dimensions and localization of conduction pathways within the TK having been reported [12–14]. Thus, understanding this variability in TK orientation is necessary to ensure successful ablation. We found that the TK orientation varies greatly between patients relative to standard RAO projections used routinely in ablation procedures while being well aligned with the direction of the CSo. Moreover, we validated this technique in vivo, with a CS-guided projection resulting in less image shortening during ablation procedures. Thus, consistent with previous studies [3], our results establish that the direction of the CSo provides a reliable landmark for identifying and optimizing TK images in patients during electrophysiology procedures.

The use of CT technology in the current study allowed us to determine anatomical TK relationships in living hearts. The TK’s size and anatomic details have been reported by Inoue et al. [15], but these date were based on autopsy. Saremi et al. reported the AV node anatomy and its blood supply using CT image in living hearts [16], and our study focused on TK orientation and its correlation with CS direction in living hearts in order to improve the X-ray projection during ablation. Hence, our findings clearly showed that the CSo direction tracks the TK orientation well, with less image shortening compared to traditional RAO projections. Furthermore, our in-vivo and during-procedure validation data indicated that the CS-guided X-ray projection can provide an image that had less shortening than those obtained from traditional RAO30 and RAO45 projections. While a CS-guided approach was optimal for the majority of patients, three outliers were found, in whom we did not get a greater distance using RAOCS. These outliers were caused by not being able to advance the CS catheter deep enough or due to abnormal CS anatomy (e.g., a location too close to the atrium in our cohort), which may have influenced the effect of using the CS catheter as a landmark to guide individual projections. Nonetheless, a CS-guided projection proved superior to traditional RAO projections to image the TK in our cohort.

The choice of projection angle is an important factor in image optimization during EP procedures. While some electrophysiologists have suggested the use of the CSo to determine the optimal, or individualized, projection angles during ablation procedures, this approach is not routinely used by every operator, possibly because no data has previously been generated in live patients. Currently, validation of CS-guided TK imaging for AVNRT ablation in living hearts, which requires precise mapping of the lower region of the TK, is lacking. Using CS-guided methods, the TK image is easier to optimize and we can clearly identify the location of the mapping catheter within the TK, which can help confirm if the mapping catheter gets close to crucial structures such as CS and His bundle. This can be achieved by adjusting the projection direction to get overlap between the descending and ascending parts of the CS catheter or the proximal electrodes of CS catheter to minimize the distances on screen, which has been indicated by electrophysiologists in the past [3, 17] and was validated in the current study. Thus, using the CS ostium direction during an ablation procedure should lead to shorter procedure times thereby minimizing X-ray exposure time while rendering procedures more safe and efficient by limiting procedural complications, and this can provide an additional projection choice for centers who are using fixed X-ray projection facing difficult cases.

There are some limitations for our study. Our study only included structurally normal hearts, which comprises the majority of AVNRT or supraventricular tachycardia patients. Clearly our studies are not immediately applicable to patients with structural abnormalities. Further, our approach is only relevant for imaging techniques involving radiation exposure and 2D projections. Accordingly, although more sophisticated 3D CT imaging modalities are highly desirable, they involve technologies that are cumbersome and time consuming, thereby being limited to small patient populations. In the meantime, in a subset of patients, abnormalities in CS anatomy and/or failure to advance the CS catheter into the distal CS may provide a misleading marker and greater image shortening. In such cases, attention must be paid in such patients to ensure image optimization.

## Conclusion

In conclusion, the triangle of Koch exhibits significant individual anatomical variability, which could influence EP procedures. The CS direction offers a reliable approach to overcome the diversity in TK orientation and assess an individualized projection during ablation to minimize the shortening of the image.

## Supplementary information

**Additional file 1: Supplementary Figure 1.** Flow chart of CT data screening.

**Additional file 2: Supplementary Figure 2.** A) The comparison of older and younger patients. The TK orientations in patients older than 45 years old is greater than TK orientations in younger ones, with statistic difference(*P* = 0.0011). B) The TK orientations in male and female patients showed no statistic difference.

## Data Availability

The datasets used and analyzed during the current study are available from the corresponding author on reasonable request.

## Abbreviations

TK: Triangle of Koch
AV: Atrioventricular
AVN: Atrioventricular node
AVNRT: Atrioventricular node reentry tachycardia
EP: Electrophysiology
RAO: Right anterior oblique
CSo: Coronary sinus ostium
CT: Computed tomography
CTA: Computed tomography angiography
AO: Aorta
ER: Eutachian ridge
TVA: Tricuspid valve attachment
RAOCS: CS-guided RAO projection
ANG: Angle
