# Supplementary figures and images for: Revisiting right anterior oblique projections for the triangle of Koch: implications from computed tomography
Source: BMC Cardiovasc Disord. 2020 Aug 24;20:383. doi: 10.1186/s12872-020-01632-9 (PMC7446209; doi:10.1186/s12872-020-01632-9)

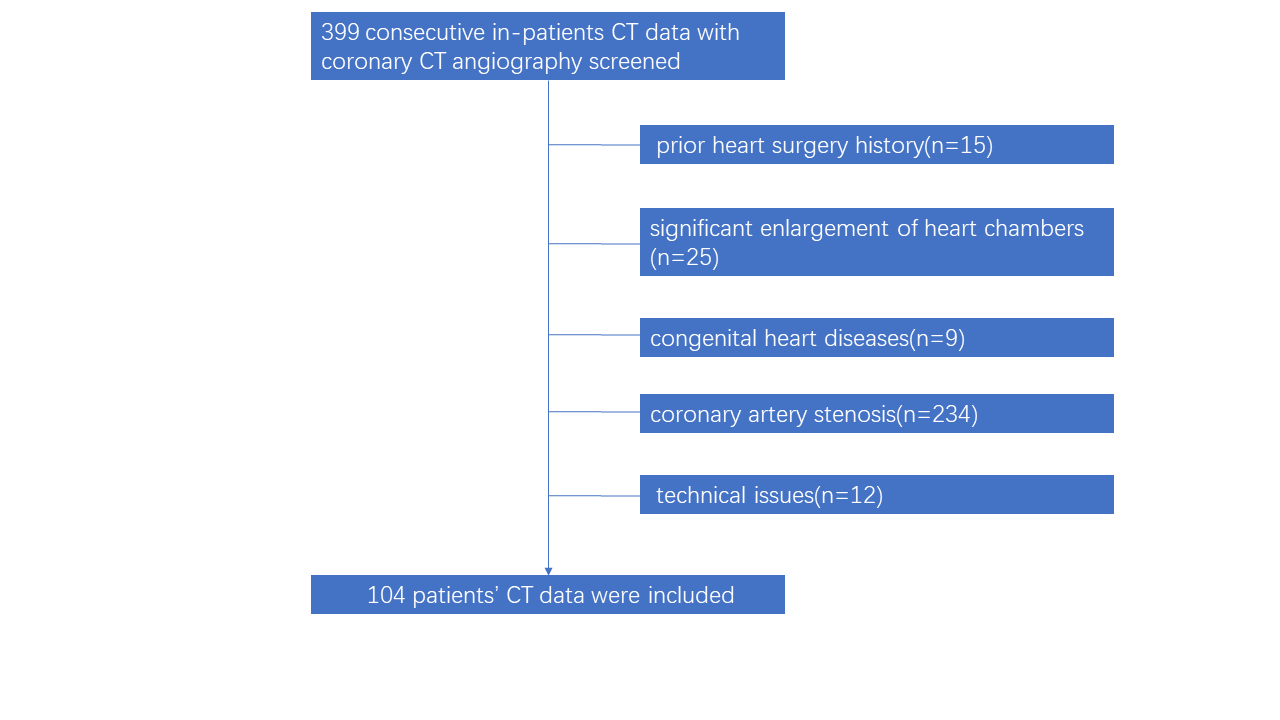

Supplement: Supplementary file 1 — Additional file 1: Supplementary Figure 1. Flow chart of CT data screening. [file 12872_2020_1632_MOESM1_ESM.tif]

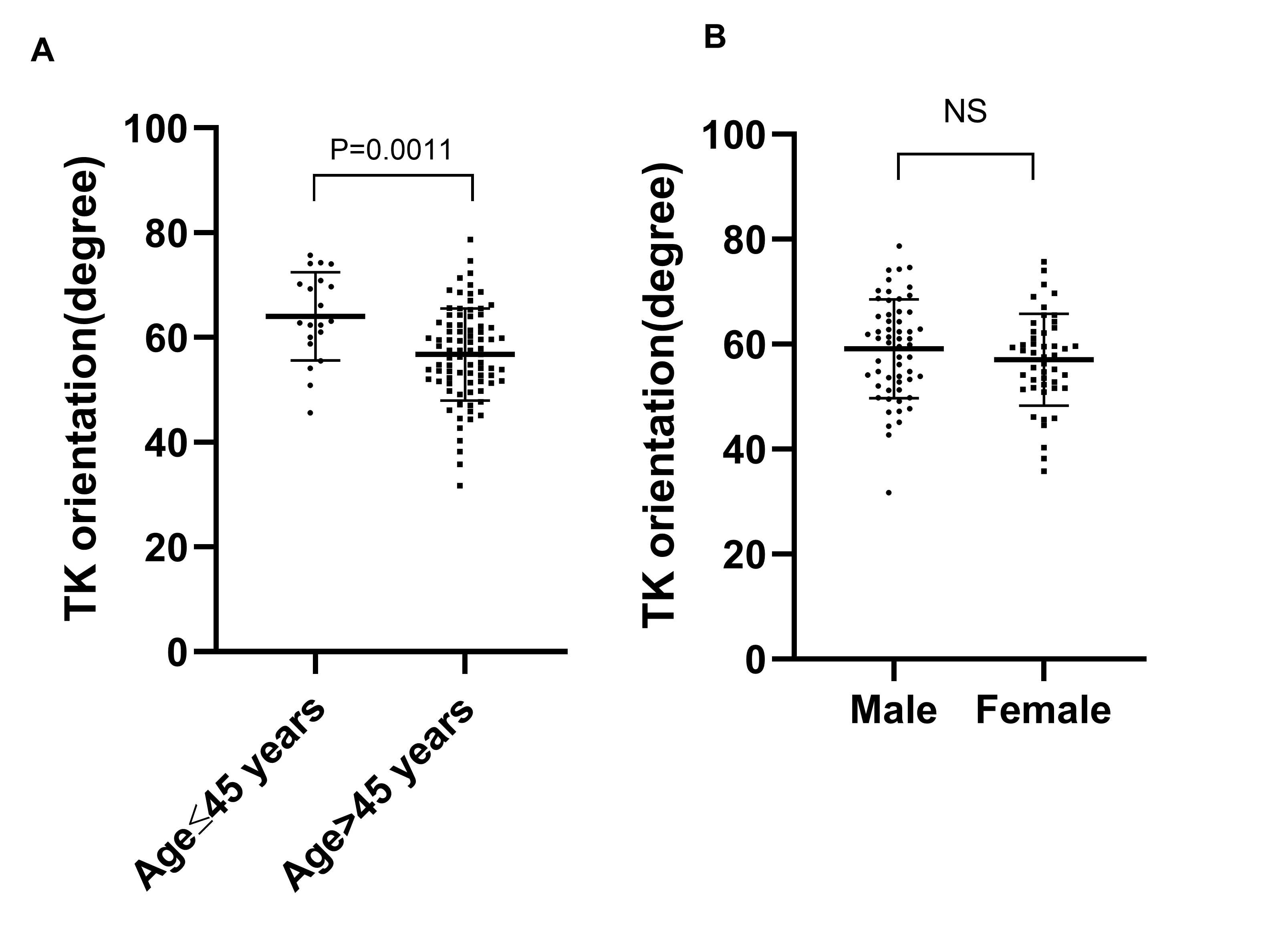

Supplement: Supplementary file 2 — Additional file 2: Supplementary Figure 2. A) The comparison of older and younger patients. The TK orientations in patients older than 45 years old is greater than TK orientations in younger ones, with statistic difference(P = 0.0011). B) The TK orientations in male and female patients showed no statistic difference. [file 12872_2020_1632_MOESM2_ESM.tif]
